# Identification of Candidate Carboxylesterases Associated With Odorant Degradation in *Holotrichia parallela* Antennae Based on Transcriptome Analysis

**DOI:** 10.3389/fphys.2021.674023

**Published:** 2021-09-10

**Authors:** Jiankun Yi, Shang Wang, Zhun Wang, Xiao Wang, Gongfeng Li, Xinxin Zhang, Yu Pan, Shiwen Zhao, Juhong Zhang, Jing-Jiang Zhou, Jun Wang, Jinghui Xi

**Affiliations:** ^1^College of Plant Science, Jilin University, Changchun, China; ^2^School of Life Science, Huizhou University, Huizhou, China; ^3^Changchun Customs Technology Center, Changchun, China; ^4^Rothamsted Research, University of Hertfordshire, Harpenden, United Kingdom

**Keywords:** *Holotrichia parallela*, antennal transcriptome, odorant-degrading enzyme, carboxylesterase, antenna-biased expression profile

## Abstract

Insects rely on their olfactory systems in antennae to recognize sex pheromones and plant volatiles in surrounding environments. Some carboxylesterases (CXEs) are odorant-degrading enzymes (ODEs), degrading odorant signals to protect the olfactory neurons against continuous excitation. However, there is no report about CXEs in *Holotrichia parallela*, one of the most major agricultural underground pests in China. In the present study, 20 candidate *CXEs* were identified based on transcriptome analysis of female and male antennae. Sequence alignments and phylogenetic analysis were performed to investigate the characterization of these candidate *CXEs*. The expression profiles of *CXEs* were compared by RT-qPCR analysis between olfactory and non-olfactory tissues of both genders. *HparCXE4, 11, 16, 17, 18, 19,* and *20* were antenna-biased expressed genes, suggesting their possible roles as ODEs. *HparCXE6, 10, 11, 13,* and *16* showed significantly higher expression profiles in male antennae, whereas *HparCXE18* was expressed more in female antennae. This study highlighted candidate CXE genes linked to odorant degradation in antennae, and provided a useful resource for further work on the *H. parallela* olfactory mechanism and selection of target genes for integrative control of *H. parallela*.

## Introduction

The insect olfactory system resides mainly in antennae and plays an integral role in mediating insect behaviors related to survival and reproduction, including locating host plants, mate partners, and oviposition sites (Younus et al., 2014; Zhang et al., 2017a). These complex olfactory behaviors rely on a series of proteins for transporting and recognizing odorant molecules, including binding proteins i.e., odorant-binding proteins (OBPs); chemosensory proteins (CSPs), chemoreceptors (i.e., olfactory receptors, ORs; ionotropic receptors, IRs), and sensory neuron membrane proteins (SNMPs; Zhou, 2010; Leal, 2013; Yi et al., 2018). However, when the odorant molecules successfully activate receptors, they must be inactivated and removed rapidly by odorant-degrading enzymes (ODEs), allowing recovery of sensitivity of the olfactory system and starting the next new potential responses (Leal, 2013; Younus et al., 2014). So far, a variety of antennal-specific and-abundant ODEs have been functionally characterized, including carboxylesterases (CXEs), cytochrome P450s (CYPs), glutathione S-transferases (GSTs), UDP-glycosyltransferases (UGTs), and alcohol dehydrogenases (ADHs), etc. (Leal, 2013; Younus et al., 2014; Zhang et al., 2017a).

CXEs belong to the α/β-fold hydrolase superfamily and include proteins implicated in neuro/developmental functions and secreted catalytically active enzymes, suggesting that they have relatively specific functions in hormone and pheromone processing and intracellular enzymic activities (Younus et al., 2014). Most esterases implicated in insecticide detoxification and metabolic resistances are intracellular enzymes, with a few secreted enzymes (Claudianos et al., 2006). CXEs commonly contain a conserved catalytic triad (Ser-His-Glu) and specifically catalyze the hydrolysis of ester bonds in various substrates (Oakeshott et al., 2005). Many insect CXEs have been identified and functionally characterized for their involvement in sex pheromone and odorant degradation to date (Sun et al., 2017). The first pheromone-degrading enzyme ApolPDE, specifically distributed in the male antennae of the moth *Antheraea polyphemus* belongs to the insect CXE family (Vogt and Riddiford, 1981). ApolPDE was later cloned and functionally characterized, and was shown to degrade the sex pheromone E6Z11-16:Ac (Vogt and Riddiford, 1981; Ishida and Leal, 2005). In the Coleoptera *Popilia japonica*, male-specific antennal esterase PjapPDE could rapidly inactivate the sex pheromone (*R*)-japonilure (Ishida and Leal, 2008). In the cotton leafworm *Spodoptera littoralis*, two esterases SlCXE7 and SlCXE10 were found to be involved in ester odorant hydrolysis, not only for sex pheromone components, Z9E11-14:Ac and Z9E12-14:Ac but also for host plant volatile (Z)-3-hexenyl acetate (Durand et al., 2010a, 2011). Among the three antennae-enriched esterases from *Spodoptera exigua*, SexiCXE4 and SexiCXE14 displayed higher degradation activities not only for ester sex pheromones, but also for ester plant volatiles (He et al., 2014a,b), while SexiCXE10 preferred to hydrolyze plant volatiles (He et al., 2015).

*Holotrichia parallela* (Motschulsky; Coleoptera: Scarabaeidae) is an economically important pest of many agricultural crops in China (Ju et al., 2012; Wang et al., 2017). Both adults and larvae could cause damage. The adults feed on the leaves, flowers, and fruits of crops, while the larvae attack the roots and other underground parts of crops, resulting in low-quality products and even plant death (Luo et al., 2009; Ju et al., 2012). Recent studies on the olfactory proteins of *H. parallela* have focused on the identification and functional characteristics of olfactory binding proteins (OBPs and CSPs; Ju et al., 2012, 2018; Fang et al., 2016), chemoreceptor proteins (ORs, IRs, and GRs; Yi et al., 2018), and microRNA (Wang et al., 2017), as well as electrophysiology and behavior bioassays (Zhou et al., 2009; Ju et al., 2017). In addition to the above studies, the morphology and distribution of antennal sensilla are also described by electron microscopy (Yi et al., 2019). Until now, very little is known about the antennal ODEs of *H. parallela*. The sex pheromone blend of *H. parallela* were also identified as two components (*L*)-isoleucine methyl ester and (*R*)-(-)-linalool (Leal et al., 1992, 1993). The previous studies have shown that males and females of *H. parallela* exhibited behavioral preferences for sex pheromones (Zhou et al., 2009) and ester plant volatiles such as (E)-2-hexenyl acetate and (Z)-3-hexenyl acetate (Ju et al., 2017). Since the sex pheromones and many odorants attracting *H. parallela* are ester molecules, it is meaningful to investigate the role of CEXs in the process of odorant degradation, and to further explore the olfactory recognition mechanism of *H. parallela*. In fact, previous studies have showed that a lot of male antennae-specific or -enriched CEXs could degrade sex pheromone components (Vogt and Riddiford, 1981; Ishida and Leal, 2005; Chertemps et al., 2012) and/or odorants (He et al., 2014a,b, 2015).

Our objective in this study was to identify candidate CXEs related to odorant degradation using male and female antennal transcriptomes and explore their putative functions. The candidate *CXEs* were identified and phylogenetic characteristics were also analyzed. In addition, the tissue expression patterns of the identified *H. parallela CXEs* were investigated in olfactory tissues (antennae) and non-olfactory tissues (heads, thoraxes, abdomens, legs, and wings) and their potential functions were predicted and discussed.

## Materials and Methods

### Insect Rearing and Extraction of Total RNA

The insects *H. parallela* were obtained from the Institute of Plant Protection, Chinese Academy of Agricultural Sciences, Beijing, China. They were reared in plastic containers (100×50cm) with damp soil (20% moisture) at 25°C, 80% RH, and on a 12-hL:12-hD photoperiod and supplied with fresh elm, *Ulmus parvifolia* Jacquin, leaves until RNA extraction (Yi et al., 2018). Various tissues, including antennae, heads, thoraxes, abdomens, legs, and wings, were dissected separately and immediately thrown in liquid nitrogen. These tissue samples were frozen at −80°C until use. Total RNAs of all tissues were extracted using TRIzol Reagent (Invitrogen, Carlsbad, CA, United States) according to the manufacturer’s protocol. The RNA integrity and purity were examined by 1.2% agarose electrophoresis and with a NanoDrop^™^ spectrophotometer (Thermo Fisher Scientific, Waltham, MA, United States).

### Construction of cDNA Library, Sequencing, and Assembly

Fifty male or female antennae were used for the RNA extraction and transcriptome analysis. The poly (A) mRNA was separated from 20μg of total RNA and purified with Oligo d(T) magnetic beads, and fragmented into short fragments by fragmentation buffer. The first-strand of cDNA was synthesized using a random hexamer primer with these mRNA fragments as templates. Next, the second-strand of cDNA was synthesized by adding DNA polymerase I, dNTPs, and RNase H and purified with a QIAquick PCR purification kit (Qiagen, Hilden, Germany), resolved with EB (ethidium bromide) buffer for end reparation and single nucleotide A (adenine) addition to the 3' end of the cDNA. Next, the short fragments were connected with sequencing adapters. After that, fragment sizes were assessed by agarose gel electrophoresis, and the appropriate fragments were subjected to PCR amplification and sequencing (Illumina HiSeq^™^ 2000, San Diego, CA, United States) by the Beijing Genomics Institute (BGI) sequencing company (Shenzhen, China). After sequencing, image deconvolution and quality value calculations were performed using the Illumina GA pipeline 1.3 (Huang et al., 2012). Then, the low quality reads (with >50% of nucleotides for which the Phred Quality Score Q was less than or equal to 5) were filtered out to generate clean reads. Transcriptome *de novo* assembly was carried out with short reads assembling program – Trinity (version 20130225;^1^ Grabherr et al., 2011). Firstly, clean reads with a certain length of overlap were combined to form longer contiguous sequences (contigs). Then the clean reads were mapped back to contigs; with paired-end approaches it was able to detect contigs from the same transcript as well as the distances between these contigs. Next, Trinity connected the contigs, and sequences that could not be extended were obtained. These result sequences of Trinity were defined as ‘unigenes’ by the BGI Company (Shenzhen, China; Xiao et al., 2015; Xie et al., 2015).

### Functional Annotation for Transcriptome Data

The unigenes were firstly aligned to protein databases using BLASTx, including those from the database of Nr, COG,^2^ Swiss-Prot,^3^ and the KEGG^4^ with a cut-off E-value of <10^−5^, and they were also aligned to the Nt database using BLASTn with a cut-off E-value of <10^−5^. With Nr annotation, Blast2GO (v2.5.0) was used to obtain gene ontology (GO) annotation of the unigenes. After getting GO annotation for every unigene, Web Gene Ontology Annotation Plot software was used to complete GO functional classification for all unigenes. With the KEGG database, the metabolic pathway annotation for unigenes was also performed. The above detailed steps are similar to a previous study (Huang et al., 2012).

### Identification of Candidate CXE Genes

Candidate CXE genes were chosen from the transcriptome data. Further, all candidate CXEs were manually checked by the BLASTx program at the National Center for Biotechnology Information (NCBI). The open reading frames (ORFs) of candidate *CXE* genes were predicted using the ORFfinder program.^5^ Then, the theoretical pI and Mw of deduced CXE proteins were calculated using the ExPASy tool with average resolution.^6^ Next, the putative N-terminal signal peptides of deduced CXE proteins were predicted using the SignalP 5.0 server, and the organism group was set to Eukarya.^7^ Multiple sequence alignment of identified CXE sequences was generated using the online Clustal Omega program.^8^

### Phylogenetic Analysis of Candidate CXE Genes

The amino acid sequences for constructing phylogenetic trees were obtained from Coleoptera, Lepidoptera, Hymenoptera, and Diptera species, including *Tribolium castaneum* (Tcas), *Leptinotarsa decemlineata* (Ldec), *Bombyx mori* (Bmor), *A. polyphemus* (Apol), *S. littoralis* (Slit), *S. exigua* (Sexi), *Spodoptera litura* (Slitu), *Drosophila melanogaster* (Dmel), *Anopheles gambiae* (Agam), and *Apis mellifera* (Amel). Their accession numbers from Genbank are listed in Supplementary Table 2. The amino acid sequences were aligned using ClustalX 2.0 software.^9^ The unrooted neighbor-joining (NJ) trees of candidate CXEs with full-length ORFs were constructed using the MEGA 7.0 software with the p-distance model.^10^ Gaps/missing data were treated as partial deletion with a site coverage cut-off=95%. Node support was assessed using a bootstrapping procedure based on 1,000 replicates. Some CXEs functionally characterized as ODEs or restrictively expressed in olfactory sensilla were marked with black dots in the phylogenetic trees from species *A. polyphemus* (Apol-PDE and Apol-ODE; Ishida and Leal, 2002, 2005), *D. melanogaster* (DmelEst6, DmelJHE and DmelJHEdup; Chertemps et al., 2012, Steiner et al., 2017, Hopkins et al., 2019), *S. exigua* (SexiCXE4, 10, 14; He et al., 2014a,b, 2015), *S. littoralis* (SlitCXE7, SlitCXE10 and Slit-EST; Durand et al., 2010a, 2011, Merlin et al., 2007), *Sesamia nonagrioides* (Snon-EST; Merlin et al., 2007), and *P. japonica* (Pjap-PDE; Ishida and Leal, 2008). The generated phylogenetic trees were colored and arranged using Figtree 1.42 software^11^ (Yi et al., 2018). CXEs were mainly divided into secreted enzyme, intracellular enzyme, and neuro-signaling enzyme clades according to the classification system of CXEs described previously (Durand et al., 2010b).

### Tissue Expression Profiles by RT-qPCR

Fifty male or female antennae and ten male or female heads, thoraxes, abdomens, legs, and wings were used as a biological sample for the RNA isolation and qPCR analysis. RT-qPCR was performed on a StepOne Plus Real-time PCR System (Applied Biosystems, Foster City, CA, United States) using SYBR Premix ExTaq II (Tli RNaseH Plus; Takara, Dalian, China). Candidate GAPDH, actin, and 18s rRNA were selected to evaluate the suitability as internal reference genes, and GAPDH had the most stable expression. So GAPDH was chosen as the reference gene for qPCR analysis. Primers of 20 *CXEs* and the reference gene (*GAPDH*) were designed using the Primer Premier 5 software, and are listed in Supplementary Table 3. The primer efficiencies of each gene were calculated and the primers with efficiency values ranging from 0.95 to 1.05 were selected for further experiments. RT-qPCR was performed under the following conditions: 30s at 95°C, 40cycles of 5s at 95°C, 10s at 55°C, and 34s at 72°C. This program was followed by a melting temperature analysis: 95°C for 15s, 60°C for 1min, increasing 0.3°C per min, and 95°C for 15s. Each reaction was run in triplicate from three biological replicates. The expression levels of these genes were calculated using the 2^-ΔΔCt^ method.

### Statistic Analysis

Significant differences were analyzed by *t*-test (between sexes) and one-way ANOVA (between different tissues), followed by Tukey’s HSD multiple comparisons test using SPSS software version 13.0. Expression profiles were created using the software Prism 6.0 (GraphPad Software, CA, United States; Yi et al., 2019). The significant differences between sexes were marked with asterisks (^*^, *p*<0.05; ^**^, *p*<0.01, NS, no differences). The expression levels among different tissues of each gender followed by the different lowercase or uppercase letters were significantly different.

## Results

### Annotation Results of *H. parallela* Antennal Transcriptome

Using the Illumina HiSeq2000 sequencing platform, 34,706 unigenes in total were obtained in the antennal transcriptomes and analyzed by searching against Nr (NCBI non-redundant protein sequences), Nt (NCBI non-redundant nucleotide database), Swiss-Prot, KEGG, COG (Cluster of Orthologous Groups of proteins), and GO databases. As to the results, significant matches were found against 18,312 (52.76%) unigenes in the Nr database, 8,524 (24.56%) unigenes in the Nt database, 14,071 (40.54%) unigenes in the Swiss-Prot database, 6,879 (19.82%) unigenes in the KEGG database, 6,508 (18.75%) unigenes in the COG database, and 8,919 (25.70%) unigenes in the GO database. A total of 19,025 (54.82%) unigenes were successfully annotated in at least one database, while 15,681 (45.18%) unigenes had no matching sequences in any of these databases (Table 1). The sequencing data were available at the NCBI Sequence Read Archive database with accession number SRP233063.

**Table 1:** Annotations of *H. parallela* unigenes in public databases.

Protein database	Number of unigene hit	Percentage (%)
Nr	18,312	52.76
Nt	8,524	24.56
Swiss-Prot	14,071	40.54
KEGG	6,879	19.82
COG	6,508	18.75
GO	8,919	25.70
Total	19,025	54.82

### Identification of Candidate CXEs

**Figure 1:**
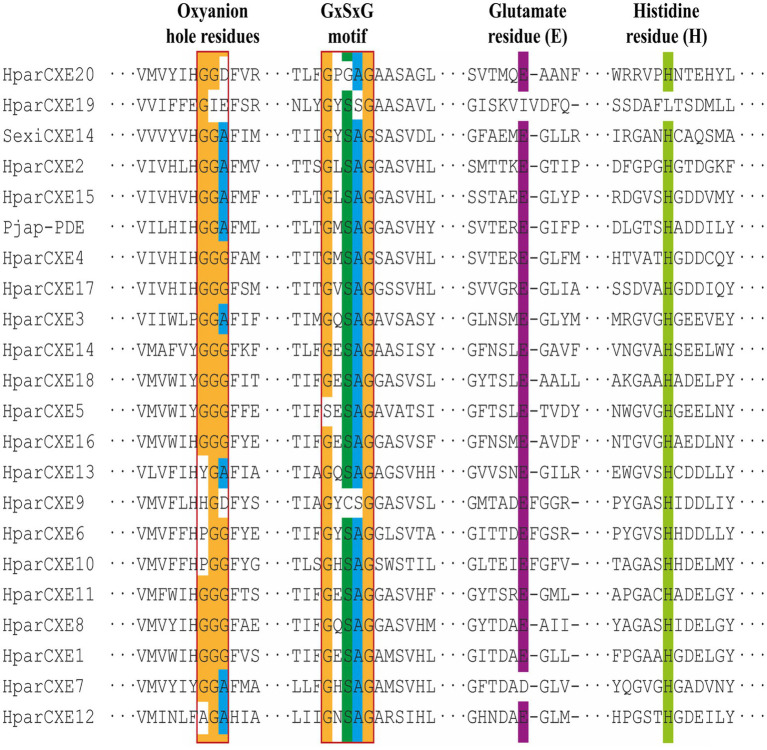
Alignments of amino acid sequences of carboxylesterases (CXEs) from *Holotrichia parallela* and other insect species. The reference amino acid sequences are from *S. exigua* (Sexi) and *Popilia japonica* (Pjap). Amino acids not shown here are represented by three sequential dots. The Gly-X-Ser-X-Gly motif and the oxyanion hole-forming residues (Gly, Gly Ala) are boxed.

In order to explore the role of CXEs in the process of odorant signal inactivation, it is necessary to identify candidate *CXEs* related to odorant degradation by blasting against the Nr database. A total of 20 candidate *CXEs* were identified with complete ORFs. The length of deduced HparCXEs was between 535 and 854 amino acid residues (Table 2). The predicted theoretical isoelectric point (pI) of HparCXEs was from 4.29 to 9.09 with the predicted molecular weight (Mw) of 60.6kDa to 98.4kDa. Furthermore, all HparCXEs except for HparCXE1, 11, and 16 were predicted to have the putative N-terminal signal peptide (SP), indicating that they could be secretory proteins. Multiple sequence alignments of candidate HparCXEs showed that they had the conserved pentapeptide motif of insect CXEs, Gly-X-Ser-X-Gly (“X” represents any residue) of typical CXE proteins except for HparCXE5, 9, and 20 (Figure 1). In addition, conserved oxyanion hole-forming residues (Gly, Gly, and Ala) were also found, which were thought to stabilize the transition states of the hydrolysis reaction (Durand et al., 2010b). The oxyanion hole-forming residues of four HparCXEs (HparCXE2, 3, 7, and 15) and nine HparCXEs (HparCXE1, 4, 5, 8, 11, 14, 16, 17, and 18) consisted of “G-G-A” and “G-G-G,” respectively (Figure 1). Additionally, the HparCXEs possessed the three conserved catalytic residues: serine (S), glutamate (E), and histidine (H), except HparCXE9 and 20 (lacking S), HparCXE7 (lacking E) and 19 (lacking E and H).

**Table 2:** Molecular characteristics and access numbers of candidate carboxylesterases.

Gene name	Access number	ORF (aa)	Mw(kDa)	PI	SP	Female FPKM	Male FPKM
HparCXE1	MN256341	554	61.82	4.29	No	7.9681	11.4349
HparCXE2	KY849880	548	61.27	5.75	Yes	149.4849	182.5306
HparCXE3	MN256342	548	62.06	6.15	Yes	65.5346	60.5936
HparCXE4	KY849884	548	61.21	5.23	Yes	257.6295	272.8335
HparCXE5	MN256343	556	62.33	6.54	Yes	1141.8355	1387.434
HparCXE6	MN256344	558	62.88	6.37	Yes	11.5945	11.6575
HparCXE7	MN256345	540	60.85	5.18	Yes	14.7613	13.5931
HparCXE8	MK863374	562	63.05	5.48	Yes	10.6951	3.9505
HparCXE9	MN256346	571	64.12	6.05	Yes	63.3226	68.1454
HparCXE10	MN256347	560	63.79	6.68	Yes	78.2333	102.1316
HparCXE11	MN256348	535	60.58	6.15	No	29.0704	40.4632
HparCXE12	MN256349	547	61.12	4.87	Yes	61.6074	78.2758
HparCXE13	MK863373	606	68.87	6.52	Yes	3.9849	16.0031
HparCXE14	MN256350	568	64.33	7.05	Yes	5.1524	4.14
HparCXE15	MN256351	568	63.89	5.84	Yes	25.9026	25.7384
HparCXE16	MN256352	555	62.74	5.28	No	47.4856	49.5223
HparCXE17	KY849897	547	60.75	5.32	Yes	29.709	21.3717
HparCXE18	MN256353	574	64.65	9.09	No	2935.9639	2625.5904
HparCXE19	MN256354	604	69.34	8.87	Yes	76.4188	57.648
HparCXE20	MN256355	854	98.36	6.32	Yes	6.4155	3.6196

### Phylogenetic Analysis of Candidate CXEs

**Figure 2:**
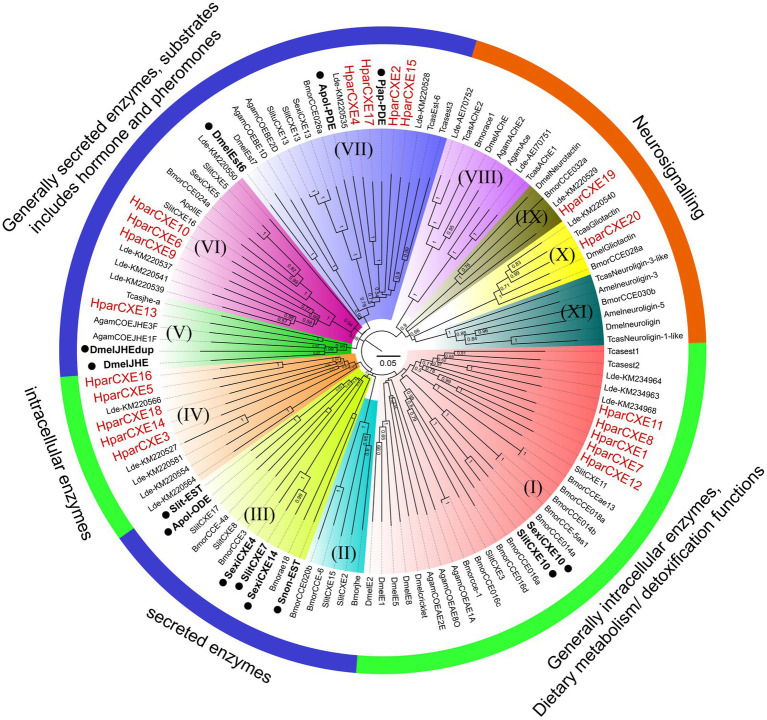
Phylogenetic analysis of candidate CXEs between *H. parallela* and other species *from Coleoptera, Lepidoptera, Hymenoptera,* and *Diptera*. The neighbor-joining (NJ) trees were constructed using MEGA7.0 software with the p-distance model. All nodes have bootstrap support based on 1,000 replicates. CXEs are divided into secreted enzymes, intracellular enzymes, and neuro-signaling enzymes. (I) α-esterase clade. (II) Lepidopteran juvenile hormone esterases. (III) Mitochondrial, cytosolic, and secreted esterases. (IV) Coleopteran xenobiotic metabolizing enzymes. (V) Juvenile hormone esterase clade. (VI) Cuticular/antennal esterases. (VII) β and pheromone esterase clade. (VIII) AchE calde. (IX) Neurotactins. (X) Gliotactins. (XI) Neuroligins. Scale bar represents the 0.05 amino acid substitutions per site. Some CXEs functionally characterized as odorant-degrading enzymes or restrictively expressed in olfactory sensilla were marked with black dots.

In order to identify the phylogenetic relationships between CXEs of *H. parallela* and those of other insect species, the NJ tree of *H. parallela* CXEs was constructed together with CXEs from several Coleoptera, Lepidoptera, and Diptera species (Figure 2). The phylogenetic analysis grouped all 20 HparCXEs into three families: the secreted enzyme family (clade II, III, V-VII: 8 HparCXEs), the intracellular enzyme family (clade I, IV: 10 HparCXEs), and the catalytically inactive, neuro-signaling family (VIII-XI: 2 HparCXEs). HparCXE1, 7, 8, 11, and 12 were clustered into the α-esterase clade in the intracellular enzyme family, well known for their involvement in detoxification of insecticide/xenobiotics and digestion of food esters (Durand et al., 2010b). This clade included some functionally characterized ODEs, such as SlitCXE10 from *S. littoralis* (Durand et al., 2010a) and SexiCXE10 from *S. exigua* (He et al., 2015). HparCXE13 shared a close relationship with juvenile hormone esterases (JHEs) of other species in the secreted enzyme family clade, with more than 90% bootstrapping support with JHEs of other species. In this clade, DmelJHE and DmelJHEdup have been functionally characterized and have high degrading activities against ester plant volatiles (Steiner et al., 2017; Hopkins et al., 2019). HparCXE6, 9, and 10 were clustered into the cuticular/antennal esterase clade in the secreted enzyme family. HparCXE2, 4, 15, and 17 were clustered into the β and pheromone esterase clade in the secreted enzyme family, together with three functionally characterized ODEs, Apol-PDE (Ishida and Leal, 2005), Pjap-PDE (Ishida and Leal, 2008), and DmelEst6 (Chertemps et al., 2012). HparCXE3, 5, 14, 16, and 18 belonged to coleopteran xenobiotic metabolizing enzymes in the intracellular enzyme family. Only HparCXE19 and HparCXE20 were clustered into the neuro-signaling enzyme family (Figure 2).

### Tissue Expression Analysis for CXEs

**Figure 3:**
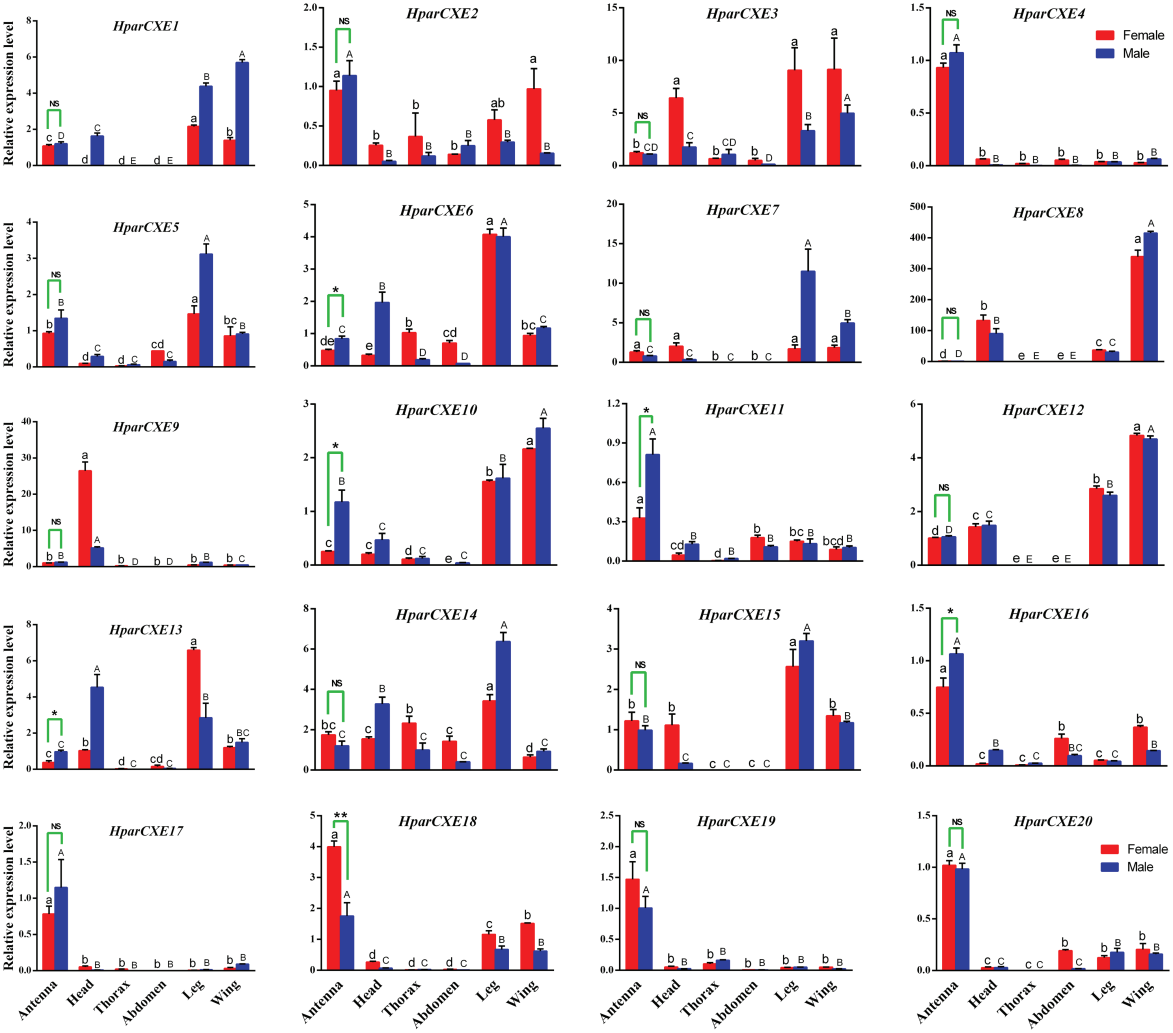
Real-time quantitative PCR validation of CXE expression levels between different sexes and tissues. Different asterisks indicate significant differences between male and female antennae (*t*-test, ^*^, *p*<0.05; ^**^, *p*<0.01; NS, no statistical significance). The uppercase and lowercase letters represent the significant differences among different male and female tissues, respectively, using one-way ANOVA.

Tissue expression profiles of all 20 *CXEs* were determined by RT-qPCR analysis (Figure 3). Five putative *HparCXEs* (*HparCXE6, 10, 11, 13,* and *16*) were significantly expressed higher in the male antennae than in the female antennae, whereas only one *HparCXE* (*HparCXE18*) showed significantly higher expression in the female antennae than in the male antennae. *HparCXE4, 11, 16, 17, 18, 19,* and *20* were expressed higher in both male and female antennae than in other tissues, whereas other *HparCXEs* were widely distributed in heads, thoraxes, abdomens, legs, and wings. *HparCXE5, 6, 14,* and *15* were expressed at a higher level in legs than in other tissues in both sexes. *HparCXE1* and *HparCXE12* had higher expression levels in both female and male wings and legs than in other tissues. *HparCXE9* was significantly expressed in both male and female heads.

## Discussion

*H. parallela* is one of the most important underground pests, and can eat leaves of crops by adults and roots by larvae, resulting in substantial yield losses (Ju et al., 2014; Yi et al., 2018). Chemical pesticides have always been the main control measure, but their use leads to the emergence of pest resistance (Wang et al., 2017). Olfaction is the main way of chemical communication between insects and the surrounding environment (Leal, 2013). Identification of ODEs involved in the termination of odorant signals will help us further explore the olfaction mechanism of *H. parallela* and provide target genes for integrative control of *H. parallela*.

This study identified for the first time 20 antennal CXEs. Bioinformatics analysis showed they are members of the alpha/beta hydrolase fold esterase family (Huang et al., 2016). Previous studies have suggested that similar to CXEs of almost insects, HparCXEs also have three conserved motifs (Figure 1): GXSXG, E, and H residues (in the GxxHxxD/E motif; Durand et al., 2010a). The S (in GXSXG), E, and H (in GxxHxxD/E) residues are the most conserved because they collectively comprise the catalytic triad (Zhu et al., 2017). As stated in previous studies, CXEs of some moths with the same conserved residues had the function of degrading odorants (Zhang et al., 2017b).

In the phylogenetic analysis, HparCXE2, 4, 15, and 17 were strongly associated with the β and pheromone esterases clade (Figure 2) in which some have been confirmed to degrade pheromones and/or plant volatiles (like Apol-PDE for the acetate sex pheromone E6Z11-16:Ac and Pjap-PDE for the female sex pheromone (R)-japonilure; Ishida and Leal, 2005, 2008). This clade also contained DmelEst6, a male antennae-enriched CEX from *D. melanogaster* (Diptera), which has a high degradation activity against the female sex pheromone cis-vaccenyl acetate (Chertemps et al., 2012). HparCXE13 was in the clade of JHEs, required for JH degradation, and pheromone or food ester degradation (Oakeshott et al., 2005; Durand et al., 2010b). In this same clade, DmelJHE in *D. melanogaster* shows higher activity with methyl decanoate and some other esters, like propyl propionate and octyl butyrate (Hopkins et al., 2019). Additionally, DmelJHEdup was shown to be an active antennal ODE against certain food acetates, including isoamyl acetate, ethyl butyrate, and ethyl propionate by physiological and behavioral experiments (Steiner et al., 2017). These results suggest that HparCXE13 may participate in ester odorant inactivation like DmelJHE and DmelJHEdup. In addition, HparCXE1, 7, 8, 11, and 12 were grouped in the α-esterase clade. In this clade, SlitCXE10 in *S. littoralis* was highly active to plant volatiles and two sex pheromone components (Z9E11-14:Ac and Z9E12-14:Ac; Durand et al., 2010b). SexiCXE10 in *S. exigua* had high activity specifically for ester plant volatiles with 7–10 carbon atoms, but no activity for sex pheromone components (He et al., 2015). Therefore, HparCXE1, 7, 8, 11, and 12 were strongly suggested as putative ODEs, and may play important roles in the detection of ester host plants and/or ester sex pheromone components of *H. parallela*. Taken together, the phylogenetic analysis showed that different HparCXEs could be involved in the different degradation process of sex pheromones, host plant volatiles, and/or other xenobiotics.

The RT-qPCR experiments were performed in order to identify the antennal-specific or antennal-biased CXEs that may be involved in odorant degradation in the antennae (Figure 3). *HparCXE4, 11, 16, 17, 18, 19,* and *20* were expressed more in antennae than in other non-olfactory tissues. Just like *jhedup*, a CEX in Drosophila, which detected food odorants and showed a predominant expression in the antennae rather than other body parts, like legs (Steiner et al., 2017). These antennae-biased expression profiles in *H. parallela* antennae suggested that these seven *HparCXEs* could play important roles in olfaction of *H. parallela* (Huang et al., 2016; Zhang et al., 2017a). *HparCXE18* presented higher expression levels in female antennae, whereas *HparCXE6, 10, 11, 13,* and *16* showed significantly higher expressions in male antennae, suggesting that the former were more related to the degradation of host plant volatiles, while the latter were more likely to participate in the degradation of sex pheromones (Yi et al., 2018). Previous studies have reported that some male antennae-specific or -enriched CEXs were shown to participate in the termination of female sex pheromones, like Esterase-6 in *Drosophila melanogaster* (Diptera; Chertemps et al., 2012) and ApolPDE in *A. polyphemus* (Lepidoptera; Vogt and Riddiford, 1981; Ishida and Leal, 2005). *HparCXE6, 10, 12,* and *15* were expressed more in legs or wings than in antennae (Figure 3), suggesting that they might not perform as ODEs, but might be involved in other physiological processes (He et al., 2014a, Zhang et al., 2017b).

The results presented here provided candidate CXEs related to odorant degradation. They need further functional verification to characterize the respective physiological roles of CXEs between sexes and in olfactory tissues. This study will provide insights in understanding the olfactory mechanism of *H. parallela* antennae and candidate target genes for integrative control of *H. parallela*.

## Data Availability Statement

The datasets presented in this study can be found in online repositories. The names of the repository/repositories and accession number(s) can be found at: https://www.ncbi.nlm.nih.gov/, PRJNA591176.

## Author Contributions

JX, JW, and JZ designed the experiment. JY performed the transcriptome experiments, organized data, and wrote this manuscript. SW performed the sequence alignments and RT-qPCR experiments. GL and ZW operated these instruments and participated in the statistical analysis. XW, XZ, YP, and SZ reared the insects and prepared the experimental samples. JJZ discussed and corrected the final manuscript. All authors contributed to the article and approved the submitted version.

## Funding

This work was supported by the project of disciplinary crossing and integration from Jilin University (JLUXKJC2020107) and the National Key Research and Development Program of China (Grant no. 2018YFD0201000 and 2017YFD0200600), and was also supported by the Professorial and Doctoral Scientific Research Foundation of Huizhou University (2020JB069).

## Conflict of Interest

The authors declare that the research was conducted in the absence of any commercial or financial relationships that could be construed as a potential conflict of interest.

## Publisher’s Note

All claims expressed in this article are solely those of the authors and do not necessarily represent those of their affiliated organizations, or those of the publisher, the editors and the reviewers. Any product that may be evaluated in this article, or claim that may be made by its manufacturer, is not guaranteed or endorsed by the publisher.

## Footnotes

^1^ http://trinityrnaseq.github.io/

^2^ http://clovr.org/docs/clusters-of-orthologous-groups-cogs/

^3^ http://www.ebi.ac.uk/uniprot

^4^ http://www.genome.jp/kegg/

^5^ https://www.ncbi.nlm.nih.gov/orffinder/

^6^ http://web.expasy.org/compute_pi/

^7^ http://www.cbs.dtu.dk/services/SignalP/

^8^ https://www.ebi.ac.uk/Tools/msa/clustalo/

^9^ http://www.clustal.org/clustal2/

^10^ http://www.megasoftware.net/

^11^ http://tree.bio.ed.ac.uk/software/figtree/
