# Supplementary material for: Identification of Candidate Carboxylesterases Associated With Odorant Degradation in Holotrichia parallela Antennae Based on Transcriptome Analysis
Source: Front Physiol. 2021 Sep 10;12:674023. doi: 10.3389/fphys.2021.674023 (PMC8461172; doi:10.3389/fphys.2021.674023)
Supplement: Supplementary file 1 [file Table_1.DOCX]

**Supplementary Table 1. Conserved motifs and BLAST results of the *H. parallela* antennal CXEs**

| **Gene name** | **Catalytic motifs** | | | | **Pfam** | | **BLASTX best hit** | | | | |
| --- | --- | --- | --- | --- | --- | --- | --- | --- | --- | --- | --- |
|  | **GxSxGx version** | **E** | **H** | **Oxyanion hole version** | **Conserved domain** | **E-value** | **Discription** | **cover** | **value** | **identy** | **Accession** |
| HparCXE1 | GESAG | + | + | GGA | COesterase | 9.80E-125 | hydrolase [Oryctes borbonicus] | 91% | 2.00E-167 | 48% | KRT83147.1 |
| HparCXE2 | GLSAG | + | + | GGA | COesterase | 1.90E-117 | hydrolase [Oryctes borbonicus] | 74% | 0 | 54% | KRT80243.1 |
| HparCXE3 | GQSAG | + | + | GGA | COesterase | 8.70E-119 | liver carboxylesterase 2-like isoform X1 [Onthophagus taurus] | 89% | 0 | 53% | XP_022916274.1 |
| HparCXE4 | GMSAG | + | + | GGA | COesterase | 3.20E-126 | esterase [Oryctes borbonicus] | 85% | 0 | 56% | KRT82414.1 |
| HparCXE5 | SESAG | + | + | GGA | COesterase | 1.60E-116 | carboxylesterase 1C-like [Onthophagus taurus] | 81% | 1.00E-132 | 41% | XP_022915842.1 |
| HparCXE6 | GYSAG | + | + | PGA | COesterase | 2.40E-132 | esterase FE4-like [Onthophagus taurus] | 83% | 0 | 56% | XP_022903453.1 |
| HparCXE7 | GHSAG | D | + | GGA | COesterase | 2.50E-124 | hydrolase [Oryctes borbonicus] | 84% | 1.00E-131 | 42% | KRT83147.1 |
| HparCXE8 | GQSAG | + | + | GGA | COesterase | 1.40E-125 | hydrolase [Oryctes borbonicus] | 79% | 0 | 61% | KRT83239.1 |
| HparCXE9 | GYCSG | + | + | HGS | COesterase | 1.10E-140 | esterase [Oryctes borbonicus] | 78% | 0 | 75% | KRT81773.1 |
| HparCXE10 | GHSAG | + | + | PGA | COesterase | 1.00E-131 | venom carboxylesterase-6 isoform X1 [Anoplophora glabripennis] | 93% | 0 | 53% | XP_018572491.1 |
| HparCXE11 | GESAG | + | + | GGA | COesterase | 1.40E-136 | esterase B1-like [Onthophagus taurus] | 85% | 0 | 58% | XP_022906488.1 |
| HparCXE12 | GNSAG | + | + | AGA | COesterase | 3.00E-115 | hydrolase [Oryctes borbonicus] | 85% | 4.00E-145 | 43% | KRT83147.1 |
| HparCXE13 | GQSAG | + | + | YGA | COesterase | 1.60E-129 | hydrolase [Oryctes borbonicus] | 84% | 0 | 52% | KRT80422.1 |
| HparCXE14 | GESAG | + | + | GGA | COesterase | 5.30E-126 | juvenile hormone esterase-like [Onthophagus taurus] | 94% | 0 | 73% | XP_022900306.1 |
| HparCXE15 | GLSAG | + | + | GGA | COesterase | 2.00E-131 | venom carboxylesterase-6-like [Onthophagus taurus] | 67% | 0 | 54% | XP_022901990.1 |
| HparCXE16 | GESAG | + | + | GGA | COesterase | 2.70E-126 | carboxylesterase 1C-like [Onthophagus taurus] | 87% | 7.00E-154 | 44% | XP_022915842.1 |
| HparCXE17 | GVSAG | + | + | GGA | COesterase | 8.70E-131 | pheromone-degrading enzyme [Popillia japonica] | 83% | 0 | 54% | AAX58713.1 |
| HparCXE18 | GESAG | + | + | GGA | COesterase | 1.40E-133 | juvenile hormone esterase isoform B [Harmonia axyridis] | 83% | 5.00E-142 | 45% | BAE16976.1 |
| HparCXE19 | GYSSG | I | L | GIS | COesterase | 3.60E-112 | carboxylesterase 4A [Onthophagus taurus] | 89% | 0 | 60% | XP_022901143.1 |
| HparCXE20 | GPGAG | + | G | GGA | COesterase | 1.50E-122 | cholinesterase isoform X1 [Onthophagus taurus] | 91% | 0% | 74% | XP_022901162.1 |
