# Supplementary material for: Identification of Candidate Carboxylesterases Associated With Odorant Degradation in Holotrichia parallela Antennae Based on Transcriptome Analysis
Source: Front Physiol. 2021 Sep 10;12:674023. doi: 10.3389/fphys.2021.674023 (PMC8461172; doi:10.3389/fphys.2021.674023)
Supplement: Supplementary file 3 [file Table_3.DOCX]

Supplementary Table 3. Primers of candidate carboxylesterases for qRT-PCR analysis

| Gene Name | Forward primer sequences (5'-3') | Reverse primer sequences (5'-3') |
| --- | --- | --- |
| *HparCXE1* | GCAACAAACAGAGATGGAGGG | ATAAACAATCTTCCTGCCCGT |
| *HparCXE2* | TGGACGGGCGTATGGTATC | TCTTTTTCTGGAACCCTTGCT |
| *HparCXE3* | AACAGGGATGCGTTTCTCAAT | GGAGTCATACAGCAGAGCGGA |
| *HparCXE4* | CAAGACCACCTGTAGGAGAA | GTTGTCACTTGGGCTTGG |
| *HparCXE5* | AACACTGCCGAAATAACGCTT | TTGATTGGTTTCACATCCCTG |
| *HparCXE6* | ATTCCCAACCAAACCTATG | AACCAATGCTGTGCTGAT |
| *HparCXE7* | CATCGCCTGCCATAAAAGC | ACCGAAGAACTGCCACAATG |
| *HparCXE8* | ATTCATTGCTTGCCCATTCC | GCGGCGTGTACTTCGTCTT |
| *HparCXE9* | CGCCCAAAGGTGTGGTGAT | CACTGTTCTTCGGTGTGGCT |
| *HparCXE10* | GATTTGCGGAAGTAGGCG | TGACAGGGTGCTCGGTAA |
| *HparCXE11* | TGGGGTCAAGTCAACGGTG | CTTTGTGATACCTGCCCTGC |
| *HparCXE12* | ATCAAATCCGAATCAGACGCT | GCTGCCTCTTCATTCCTCAAT |
| *HparCXE13* | CAGGAGGTTCTTCTGTGGCA | TCGCAGCATTCAAGGGATT |
| *HparCXE14* | AAATGGTCTGGAGTCCTGAGC | TGTCCGAACTTGAATCCGC |
| *HparCXE15* | TCGGGGGAAATCCTGAATC | AAGGTGCTAAAGCGTTGCC |
| *HparCXE16* | GCTATTGCGACCAAAACT | GCACTTGCTGTTATGGTATG |
| *HparCXE17* | ACAGGGGTGTCTCGCAAAG | TCCATCGTGTTCTGGTTCG |
| *HparCXE18* | TACATTCCGCAGAAGCCAGA | AGACCATCAGGACCGTAAAGG |
| *HparCXE19* | TCCATAGCCAGTTCTTGTCCG | GTTGGAACGGAACCCTGGA |
| *HparCXE20* | GGCAATCCATCTTACACGCA | GGCTCCCACCAAAACTCAGT |
| *GADPH* | AATACCTTTTAGTGGTCCTTCCG | TGCATGCTATCACAGCTACGC |
